# Immunoinformatics approaches to explore *Helicobacter Pylori* proteome (Virulence Factors) to design B and T cell multi-epitope subunit vaccine

**DOI:** 10.1038/s41598-019-49354-z

**Published:** 2019-09-16

**Authors:** Mazhar Khan, Shahzeb Khan, Asim Ali, Hameed Akbar, Abrar Mohammad Sayaf, Abbas Khan, Dong-Qing Wei

**Affiliations:** 1 The CAS Key Laboratory of Innate Immunity and Chronic Diseases, Hefei National Laboratory for Physical Sciences at Microscale, School of Life Sciences, CAS Center for Excellence in Molecular Cell Science, University of Science and Technology of China (USTC), Collaborative Innovation Center of Genetics and Development, Hefei, 230027 Anhui China; 2 Centre for Biotechnology and Microbiology, University of Swat, Swat, Khyber Pakhtunkhwa Pakistan; 3 Department of Bioinformatics and Biological Statistics, School of Life Sciences and Biotechnology, Shanghai Jiao Tong University, Shanghai, 200240 P.R. China; 4 Laboratory of Cellular Dynamics, School of Life Sciences, University of Science and Technology of China (USTC), Anhui Sheng, P.R. China

**Keywords:** Protein vaccines, Peptide vaccines

## Abstract

*Helicobacter Pylori* is a known causal agent of gastric malignancies and peptic ulcers. The extremophile nature of this bacterium is protecting it from designing a potent drug against it. Therefore, the use of computational approaches to design antigenic, stable and safe vaccine against this pathogen could help to control the infections associated with it. Therefore, in this study, we used multiple immunoinformatics approaches along with other computational approaches to design a multi-epitopes subunit vaccine against *H*. *Pylori*. A total of 7 CTL and 12 HTL antigenic epitopes based on c-terminal cleavage and MHC binding scores were predicted from the four selected proteins (CagA, OipA, GroEL and cagA). The predicted epitopes were joined by AYY and GPGPG linkers. Β-defensins adjuvant was added to the N-terminus of the vaccine. For validation, immunogenicity, allergenicity and physiochemical analysis were conducted. The designed vaccine is likely antigenic in nature and produced robust and substantial interactions with Toll-like receptors (TLR-2, 4, 5, and 9). The vaccine developed was also subjected to an *in silico* cloning and immune response prediction model, which verified its efficiency of expression and the immune system provoking response. These analyses indicate that the suggested vaccine may produce particular immune responses against *H. pylori*, but laboratory validation is needed to verify the safety and immunogenicity status of the suggested vaccine design.

## Introduction

*Helicobacter pylori* causes multiple infections, such as mucosal lymph tissue, peptic ulcers and gastric carcinoma^1–3^. *H*. *pylori* is mainly associated with gastric infections, along with other diseases. The risk factors for gastric cancer are probably the interaction between host and pathogen virulence factors^4^. Pathogenic factors of *H*. *pylori* cause chronic inflammation, resulting in tissues narcosis, lesions and subsequently stomach cancer^5–8^. *H. Pylori* related infections are more frequently correlated with multiple known disease causing factors. Not all but a paramount number of these factors are believed to increase the threat of infection and thus upturns the production of pro-inflammatory cytokines. Predominant stomach disorders and virulent proteins are linked with *H*. *pylori* infection, among them the widely known virulence factors of *H*. *pylori* are, Cytotoxin associated genes (Cag), the Pathogenicity island (PaI), and are thought to play crucial role in eliciting inflammation^9^. According to available scientific studies, comparing the cagA strains having very few phosphorylated motifs to those having phosphorylation in abundance, the later are more involved in gastric carcinoma^10–12^, while OipA is mainly involved in duodenal ulcers^13,14^. Further, human gastric biopsy samples revealed the involvement of OipA^15,16^ and cagA in gastric cancer and duodenal ulceration^17,18^.

Numerous and drastic effects of the VacA on epithelial cells pave a way to gastric cancer. The increased risk of cancer is correlated with the active form of VacA^19^. The innate immune response is principally dependent on the pattern recognition receptors recognizing the pathogen-associated molecular patterns of the pathogen^20^. The pattern recognition receptor Nod1 is an intracellular receptor; it recognizes the PAMP and is stimulated by CagA strains. Toll-like receptors are the most studied pathogen recognition receptors (PRRs), TLRs family comprises of eleven proteins, and each of them uniquely binds to different PAMPs, they are expressed on surface of the cells^21^. According to the transfected cell line studies, *H*. *Pylori* elicits the expression of pro-inflammatory genes via different receptors (TLR2, TLR4, TLR5 and TLR9)^22–25^. High level of Alpha 1, 2 and 3 defensins along with β-defensins are quantified in infections caused by *H*. *pylori* such as intestinal cancer. Higher fold expression can be obtained via using these defensins in *in-silico* cloning.

The pathogenic role of these proteins in *H*. *pylori* infections makes them more ideal factors for vaccine designing, which will provide long term immunity against *H*. *pylori* infections. A potent vaccine against the *H*. *pylori* infection has not yet designed. Literatures on the mechanism of interactions between *H*. *pylori* and human immune responses are available that can be exploited for designing of a potent vaccine^26^. Epitopes are the antigenic portions of the pathogens that are recognized by host immune system and humoral or cell mediated immunity is triggered against it^27^. Immediately after infection antigen-presenting cells (APCs) activates the cytotoxic T lymphocytes, that have dominant role in killing the infected cells^28^. Peptides bound to MHC acts on surface of *H*. *pylori* infected cells^29^. Hence, a wide ranges of high binding affinity epitopes are displayed by MHC molecules.

Recent advance in the field of immunoinformatics and the availability of diverse array of tools to design epitope vaccines has profoundly increased the research on the development of novel potential vaccine candidates. The use immunoinformatics approaches and tools are reasonably effective for the development of precise and stable multi-epitope subunit vaccine^30^. Immunoinformatics is accurate, reliable and rapid approach for developing vaccine against virulent pathogens. Taking into account the feasibility and advantages of the vaccine designed through immunoinformatics approaches, this study also aims to design a multi-epitopes subunit vaccine against *H*. *pylori*. Herein, we used multiple antigenic proteins from *H*. *pylori* proteome to design B and T-cell epitopes from them. Prediction of HTL epitope was performed and the final vaccine composed of multiple epitopes was constructed. We further used molecular docking, thermodynamics stability profiling, *in-silico* expression and an agent-based modeling tool to verify the stability, expression and immune response reaction provoked by the final vaccine.

## Methodology

### Collection of proteins

**Figure 1:**
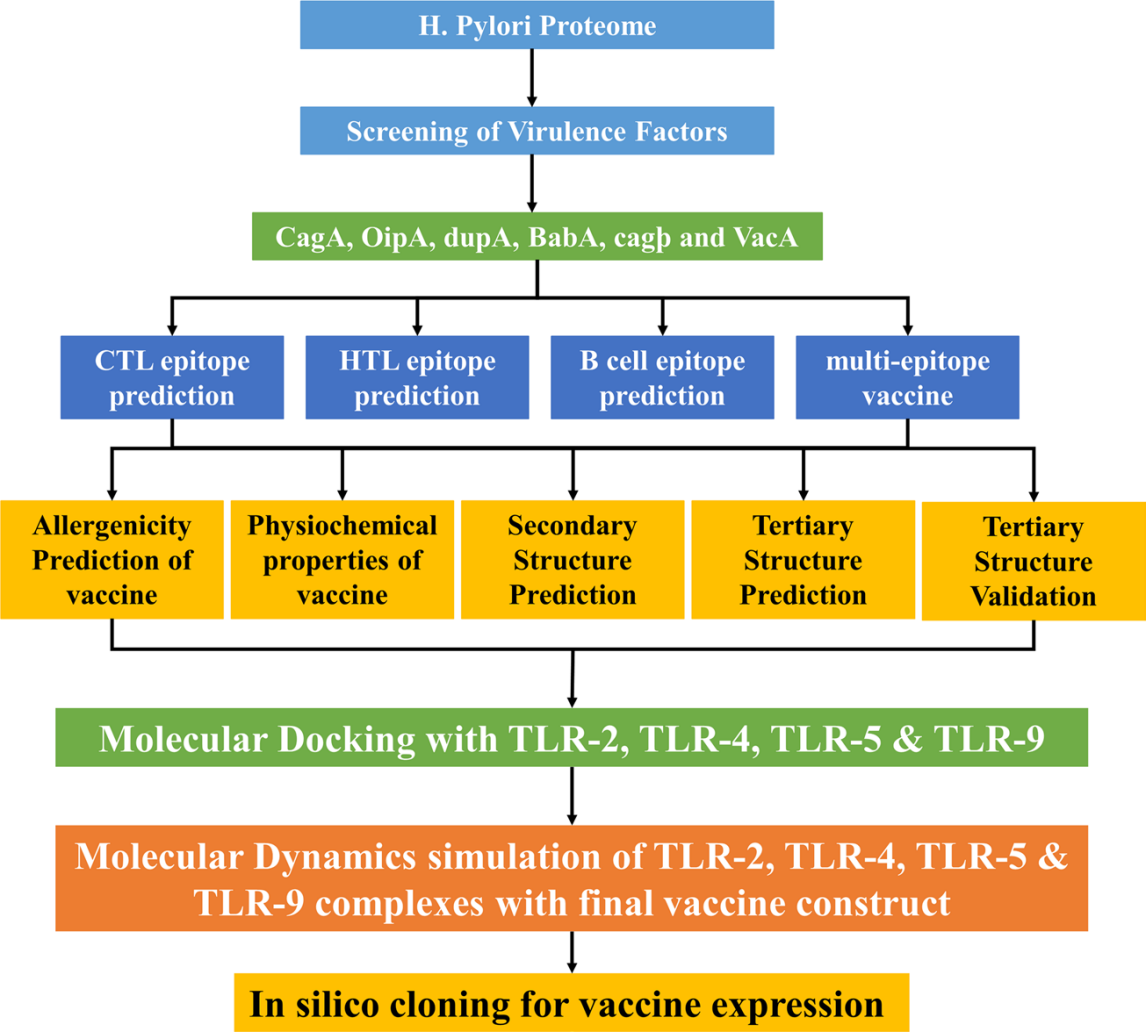
Workflow of this scientific study. The complete methodology is shown is four steps: the selection of proteins suitable for vaccine designing, the second step comprised of T and B cell epitopes prediction, construction of vaccine by joining together the epitopes, at last, molecular docking with TLRs and MD simulations for stability of complexes and finally *in silico* expression was performed.

Four proteins were selected for vaccine designing against *H*. *pylori*, including CagA, OipA, GroEL and VacA based on their role in *H*. *pylori* infection. Proteins that are important for virulence of a pathogen are suitable for designing a vaccine. The workflow of this scientific study is shown in Fig. 1.

### MHC-I binding epitopes (CTL) prediction

CTL epitopes were predicted for four virulent proteins (VacA, GroEL OipA, and CagA,) of *H*. *pylori* using NetCTL1.2 (http://www.cbs.dtu.dk/services/NetCTL/)^31^, an online tool. The server predicts 9-mer epitopes for the query protein based on transport-associated protein, C-terminal cleavage scores and MHC-I binding score. Artificial neural network is employed by the server for C-terminal cleavage and binding of peptides to MHC-I whereas Tap transport efficiency is obtained by the weight matrix. The epitopes were predicted at 0.75(default) threshold.

### MHC-II binding epitopes (HTL) prediction

MHC-II binding, 15-mer epitopes, were predicted by a web tool IEDB MHC-II for each of the selected virulent proteins (CagA, GroEL, OipA, and VacA) against a set of seven human HLAs given as;, HLA-DRB1 * 15:01, HLA-DRB4 * 01:01, HLA-DRB3 * 01:01, HLA-DRB5*01:01, HLA-DRB1 * 03:01 HLA-DRB3 * 02:02, HLA-DRB1 * 07:01. The server defines the affinity of peptides towards the MHC-II on the basis of IC_50_ score. IC_50_ values less than 50 nM shows highest binding affinity towards MHC-II, IC_50_ less than 500 nM indicates midrange affinity whereas IC_50_ less than 5000 nM corresponds to lowest binding affinity. The percentile rank is inversely related to the IC_50_ score.

### B-cell epitopes prediction

B lymphocytes are the most important factor of the immune system. It is responsible for secreting antibodies that in turn, provides long term immunity. For the prediction of B-cell linear epitopes, we employed an online server BCPred (http://ailab.ist.psu.edu/bcpred/)^32^, the server exploits kernel technique to predict 20-mer linear B-cell epitopes. BCPred uses a support vector machine (SVM) algorithm for B-cell (linear) epitopes prediction.

For the prediction of discontinuous B cell epitopes, we used a web server ElliPro (http://tools.iedb.org/ellipro/). Residues clustering algorithms along with Thornton’s technique is employed by ElliPro suite for prediction of B-cell (conformational) epitopes. The server exploits Modeler v9.20 in order to create 3D coordinates of the epitopes predicted by the server. The server assigns a PI (protrusion index) value to each predicted epitope^33^.

### Construction of vaccine sequence

A set of CTL and HTL epitopes were selected on the basis of its high binding score and non-allergenic nature. In order to make the final multi-epitopes vaccine construct, the selected CTL epitopes were linked together by AAY linker whereas GPGPG linkers were used for HTL epitopes. Epitopes representation and proper separation are enhanced by linkers. The linkers are significant for two reasons; (1) linkers effectively prevent the formation of neo-epitopes (junctional epitopes), (2) and improve epitope presentation^34–37^. Furthermore, in order to improve immunogenicity, human β-defensins was added at N-terminus of the vaccine using EAAAK linker as an adjuvant.

### Allergenicity profiling

To make sure the vaccine don’t elicit any allergen response we used an online antigenicity prediction server AlgPred (http://www.imtech.res.in/raghava/algpred/), the server uses several algorithms (SVMc + MAST + IgEepitope + ARPs BLAST) to check allergenicity of query sequence^38^. The server results are reliable and have 85% precision at −0.4 threshold.

### Vaccines antigenicity profiling

Antigenicity evaluation is an important step in vaccine designing. In this study, we utilized two servers for antigenicity prediction, ANTIGENpro (http://scratch.proteomics.ics.uci.edu/)^39^ and Vaxijen 2.0 (http://www.ddg-pharmfac.net/vaxijen/VaxiJen/VaxiJen.html). For antigenicity evaluation of the query sequence the former uses microarray data, it employs pathogen independent and sequence-based approach to predict the antigenicity value. However, the later predicts antigenicity on the basis of physicochemical properties for analysis of the query sequence. Vaxijen have 70 to 89% precision depending on the organism.

### Physiochemical properties evaluation

For assessment of several physicochemical properties of the vaccine sequence an online freely accessible web server ProtParam (http://web.expasy.org/protparam/)^40^ was utilized. The server calculates amino acid composition, molecular weight, aliphatic score, *in*-*vitro* half-life, *in vivo* half-life, instability index, theoretical pI, and GRAVY.

### Prediction of secondary structure

For the computation of secondary structure, we used a web server PSIPRED (http://bioinf.cs.ucl.ac.uk/psipred/)^41^ for the vaccine sequence, the server provides results with high accuracy. Proteins showing homology to our vaccine construct were identified via PSI-Blast, these sequences were then used to create PSSM (position-specific scoring matrix). Furthermore, the server uses neural network (feed forward) to process the PSSM and predict the secondary structure elements.

### Vaccine 3D structure prediction

For the prediction of 3D structure for our vaccine sequence, we employed a widely used, freely available online web server RaptorX (http://raptorx.uchicago.edu/)^42^. The server along with 3D structure prediction, can predict disordered regions, solvent accessibility, secondary structures, binding sites and contacts. RaptorX predicts the absolute global quality and comparable global quality for each of the residues of the query sequence. In order to check the 3D structure, we used Pymol software.

### Vaccine 3D structure refinement

The 3D structure was subjected to an online server Galaxy Refine (http://galaxy.seoklab.org/)^43^ for further improvement. The server employs the CASP10 technique in order to refine the query 3D structure. The side chains of proteins are reconstructed by CASP10 technique followed by repacking and use of simulations of the 3D structure for relaxation. Galaxyrefine improved the structural and global quality of the 3D structure. YASARA^44^ software was used for energy minimization and correction of the structure.

### Vaccine 3D structure validation

Three freely available web tools ProSA-web (https://prosa.services.came.sbg.ac.at/prosa.php)^45^, ERRAT (http://services.mbi.ucla.edu/ERRAT/)^46^ and RAMPAGE (http://mordred.bioc.cam.ac.uk/rapper/~rampage.php)^47^ were utilized for the refined 3D structure validation. Prosa-Web highlight and plots overall excellence scores of the errors calculated in the query 3D structure, ERRAT server focus on the nonbonded interactions within the given structure whereas RAMPAGE server investigates Ramachandran plot, it utilizes PROCHECK principles for validation via Ramachandran plot. A separate plot is drawn for Proline-glycine residues.

### Interaction analysis vaccine with TLR receptors

For the evaluation of the interaction between the vaccine and human toll-like receptors 2, 4, 5 and 9 we employed PatchDock (http://bioinfo3d.cs.tau.ac.il/PatchDock/)^48^. It computes separating scores, surface fix coordinating scores, and portrayal of atomic shape for molecular docking. The server divides both the vaccine and TLRs into small patches in agreement with the surface shape. These small patches resemble unique shapes, which can distinguish between puzzle pieces visually. Another algorithm does superimposition of these small patches after identification of the patches. Furthermore, for refining and re-scoring the molecular docking complexes another server was employed, FireDock^49^ to get the best structure. The refined complexes provided by FireDock are based on several factors such as atomic contact energy, partial electrostatics and vdW.

### Molecular dynamics simulation

Amber 14 setup was utilized for all the complexes to perform MD simulations^50^. Addition of Sodium ions neutralized the system by using “tleap”. A TIP3P water box was used to solvate the system. To remove the clashes and constraints in the system energy minimization was carried out on AMBER version 14. The minimized system was used for MD analysis using PMEMD.cuda. A cutoff radius of 10 Å for non-bond interactions was considered. SHAKE and Particle-Mesh Ewald (PME) algorithms were utilized^51^. Post simulation analysis of the ten nanoseconds trajectories was performed using CPPTRAJ and PTRAJ^52^. Finally, RMSDs and RMSFs of all the systems were calculated.

## Validation

### Codon optimization and *in-silico* vaccine expression

Jcat was used for reverse translation and optimization of codons in order to achieve maximum expression in *E*. *coli* cellular machinery, Jcat calculated the GC content and CAI scores for the query sequence in order to ensure maximum expression. Prokaryotic ribosome and restriction binding sites and rho-independent termination options were selected. Restriction sites of Xhol and Ndel were added to the reverse translated sequence. The final vaccine was then cloned into pET-28a(+) plasmid using snap-gene software.

### Immune Simulation

C-ImmSim^53^ is an online server, which uses an agent-based modelling approach to estimate the effect of a foreign particle, antigen, on the immune system. The server reflects the immune response against the antigen using PSSM method. Antibodies, cytokines and interferon production upon the injection of the vaccine is calculated. In addition, Th1 and Th2 responses are also forecasted by the webserver. Default parameters were used to plot the Simpson Index or D (a measure of diversity).

## Results

### *Helicobacter pylori* protein sequences retrieval

The amino acid sequences of the selected proteins GroEL, OipA, CagA and VacA of *Helicobacter pylori* were retrieved from Gene Bank using GI: 446963037, GI: 446632395, GI: 2498230 and GI 15645505 to design multi-epitope subunit vaccine for immune response against *H*. *pylori* infection. The selection of these proteins are based on their target efficacy and reliability. Immunogenic behavior by vaccine was enhanced by fusing the human β-defensins 4A protein (Uniprot ID: O15263) as adjuvant, which regulates the immune response by coupling with vaccine protein.

### Antigenicity of selected *H*. *pylori* proteins

Vaxijen2.0^54^, a web server, determined the antigenicity of each protein. The results from the server showed that each protein possesses antigenic properties. Scores revealed by this server includes 0.46 for GroEL, 0.56 for OipA, 0.45 for CagA and 0.58 for VacA respectively.

### Cytotoxic and HTL epitopes prediction

A total of 72 CTL epitopes were predicted for all of the proteins by the NetCTL 1.2. Among the total 72 epitopes, only seven epitopes based on the defined criteria, MHC binding score and non-allergenic nature, were selected. Similarly, the CTL epitopes given in Supplementary Table S1 by using the IEDB MHC-II server were predicted for the selected proteins. The HTL epitopes were predicted for a set of seven HLAs (HLA-DRB1 * 03:01, HLA-DRB4 * 01:01, HLA-DRB1 * 07:01, HLA-DRB3 * 02:02, HLA-DRB1 * 15:01, HLA-DRB3 * 01:01 and HLA-DRB5 * 01:01). HTL epitopes are given in Table 1. A variable number of HTL epitopes such three from GroEL, CagA and OipA each at different positions and four from VacA were predicted by the server.

**Table 1:** Helper T-Lymphocytes epitopes are given in the table along with their scores predicted by IEDB MHC class II server.

Protein	S.NO	Allele	Start	End	Peptide sequence	method	Percentile rank
GroeL	1	HLA-DRB3*01:01	336	350	GHSHDVKDRVAQIKT	Consensus (comb.lib./smm/nn)	0.11
	2	HLA-DRB3*01:01	337	351	HSHDVKDRVAQIKTQ	Consensus (comb.lib./smm/nn)	0.11
	3	HLA-DRB1*03:01	186	200	LDVVEGMQFDRGYLS	Consensus (smm/nn/sturniolo)	0.88
oipA	1	HLA-DRB1*07:01	219	233	NLTPFNQVKSRTIFQ	Consensus (comb.lib./smm/nn)	0.01
	2	HLA-DRB1*07:01	220	234	LTPFNQVKSRTIFQL	Consensus (comb.lib./smm/nn)	0.01
	3	HLA-DRB1*07:01	191	205	KASRHVFRKSSGLVI	Consensus (comb.lib./smm/nn)	0.05
cagA	1	HLA-DRB1*03:01	806	820	SRVEQVLADLKNFSK	Consensus (smm/nn/sturniolo)	0.09
	2	HLA-DRB1*15:01	116	130	KFGDQRYQIFTSWVS	Consensus (smm/nn/sturniolo)	0.42
	3	HLA-DRB1*15:01	119	133	DQRYQIFTSWVSHQK	Consensus (smm/nn/sturniolo)	0.42
VacA	1	HLA-DRB3*02:02	1148	1162	FAFFRNALVLKPSVG	NetMHCIIpan	0.17
	2	HLA-DRB3*02:02	1149	1163	AFFRNALVLKPSVGV	NetMHCIIpan	0.5
	3	HLA-DRB5*01:01	153	167	GGDLDVNMQKATLRL	Consensus (smm/nn/sturniolo)	0.63

### Vaccine construction

**Figure 2:**
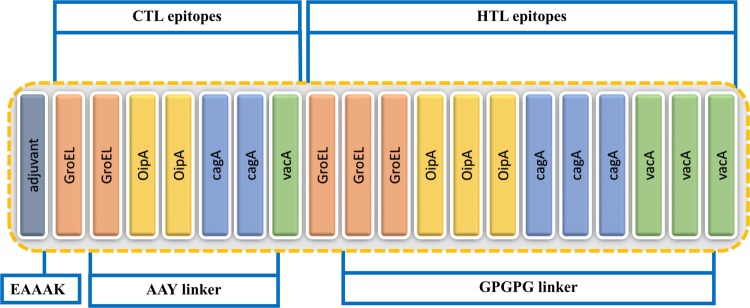
Structural arrangement of the final vaccine candidate constructed from CTL and HTL epitopes using different linkers.

Twelve HTL and seven CTL epitopes based on their binding scores, antigenic nature and the non-allergenic property is given in Table 2 were joined together by using GPGPG and AAY linkers to construct the final multi-epitopes vaccine. Adjuvant was attached to the N-terminus of the vaccine for the protection from degradation. EAAAK linker was used to join the adjuvant to the CTL epitopes. On the other hand, AAY and GPGPG linkers joined the CTL and HTL epitopes. Figure 2 is showing the final structure of the vaccine construct including. The predicted epitopes were evaluated by BLASTp to avoid epitopes homologous with human proteins. Only 16% similarity was reported which is due to the human β-defensins sequence attached at the N-terminus.

**Table 2:** A list of both HTL and CTL epitopes used to model the final vaccine structure based on their respective scores.

Sequence	Combine score	Type of Epitope
TTTATVLAY	3.34	CTL epitope
KVNYYSDDY	1.4	CTL epitope
YSDDYGDKL	2.02	CTL epitope
GISQLREEY	1.07	CTL epitope
NRDARAIAY	1.17	CTL epitope
GIDTGNGGF	0.84	CTL epitope
FASNLGMRY	2.49	CTL epitope
GHSHDVKDRVAQIKT	0.11	HTL epitope
HSHDVKDRVAQIKTQ	0.11	HTL epitope
LDVVEGMQFDRGYLS	0.88	HTL epitope
NLTPFNQVKSRTIFQ	0.01	HTL epitope
LTPFNQVKSRTIFQL	0.01	HTL epitope
KASRHVFRKSSGLVI	0.05	HTL epitope
SRVEQVLADLKNFSK	0.09	HTL epitope
KFGDQRYQIFTSWVS	0.42	HTL epitope
DQRYQIFTSWVSHQK	0.42	HTL epitope
FAFFRNALVLKPSVG	0.17	HTL epitope
AFFRNALVLKPSVGV	0.5	HTL epitope
GGDLDVNMQKATLRL	0.63	HTL epitope

These HTL and CTL epitopes sequences were used in the final structure.

### B cell epitopes prediction

**Figure 3:**
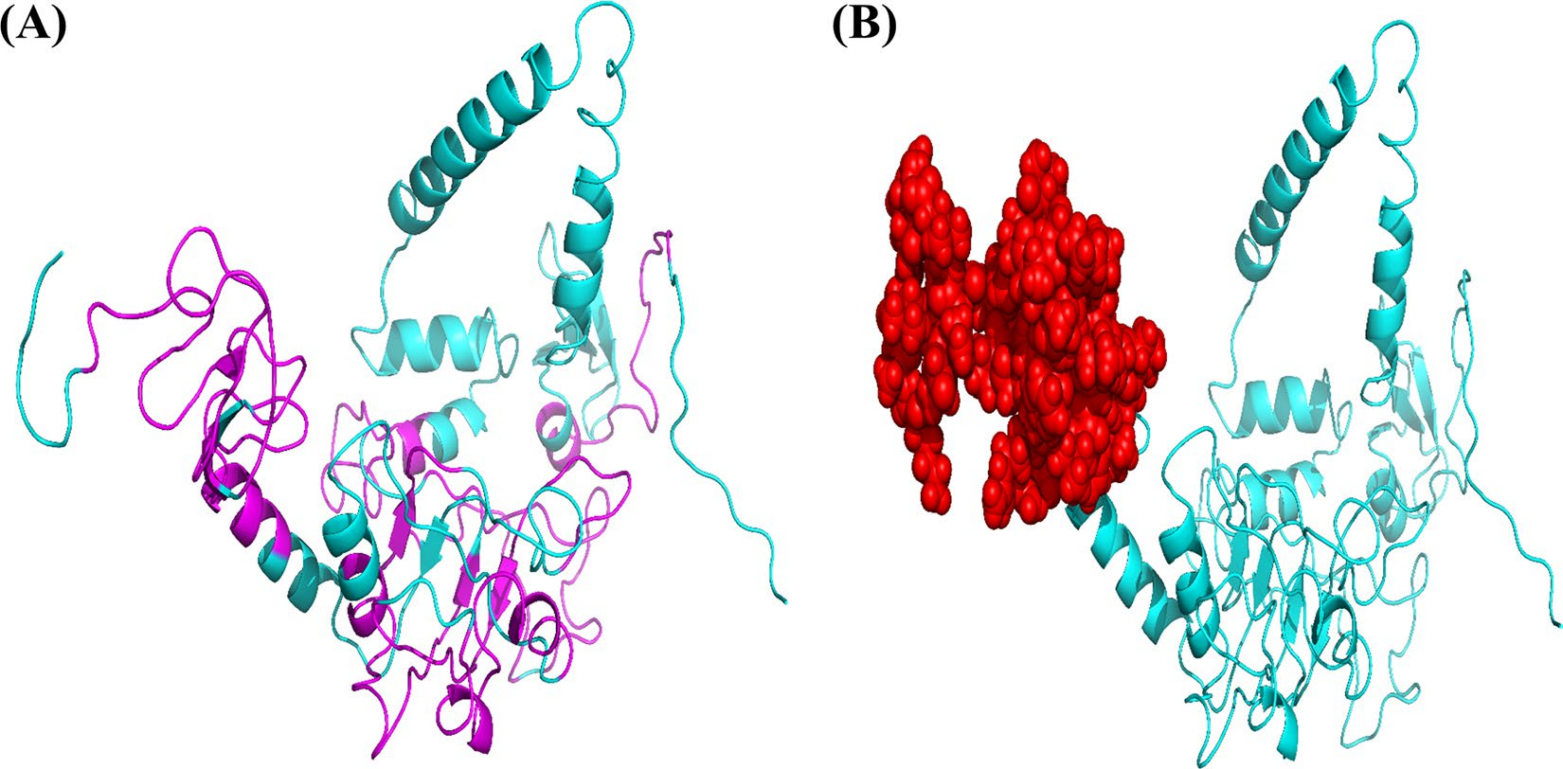
Prediction of linear B-cell epitopes by BCPred while discontinuous epitopes are predicted by ElliPro server. Panel A is showing B-cell (linear) epitopes (magenta) highlighted in vaccine 3D structure while B-cell (Conformational) epitopes are depicted in panel B (red color).

BCPred predicted 12 (linear) B-cell epitopes of 20 amino acids in length each with scores ≥0.90 were selected. Likewise, ElliPro suite predicted the discontinuous B-cell epitopes. In total, 51 amino acids were classified as B-cell (conformational) epitopes with a score of 0.819. The server default parameters were applied to predict these epitopes. The predicted conformational and linear epitopes are given in Fig. 3A,B.

### Physio-chemical properties calculations

Several essential properties such as allergenicity, molecular weight, half-life and instability were calculated. The server predicted that the final vaccine construct is non-allergenic by predicting −0.42 score. The default threshold −0.4 was used. The basic nature of the vaccine was confirmed by determining the Isoelectric point. PI score 9.64 with 41.25 kDa molecular weight (MW) was calculated by the server. On the other hand, half-life >30, >20 and >10 hours, *in vitro* (in mammal’s reticulocyte) yeast *E*. *coli* (*in vivo*) was calculated. Instability index of 17.28 confirmed that the vaccine would be stable in the experimental setup. GRAVY was calculated as −0.261, while the aliphatic coefficient was 68.28. In addition, online servers ANTIGENpro and VaxiJen have calculated 0.68 and 0.93 antigenic scores for the vaccine design, suggesting that the vaccine is immunogenic and can trigger an adequate immune effect.

### Secondary structure elements prediction

**Figure 4:**
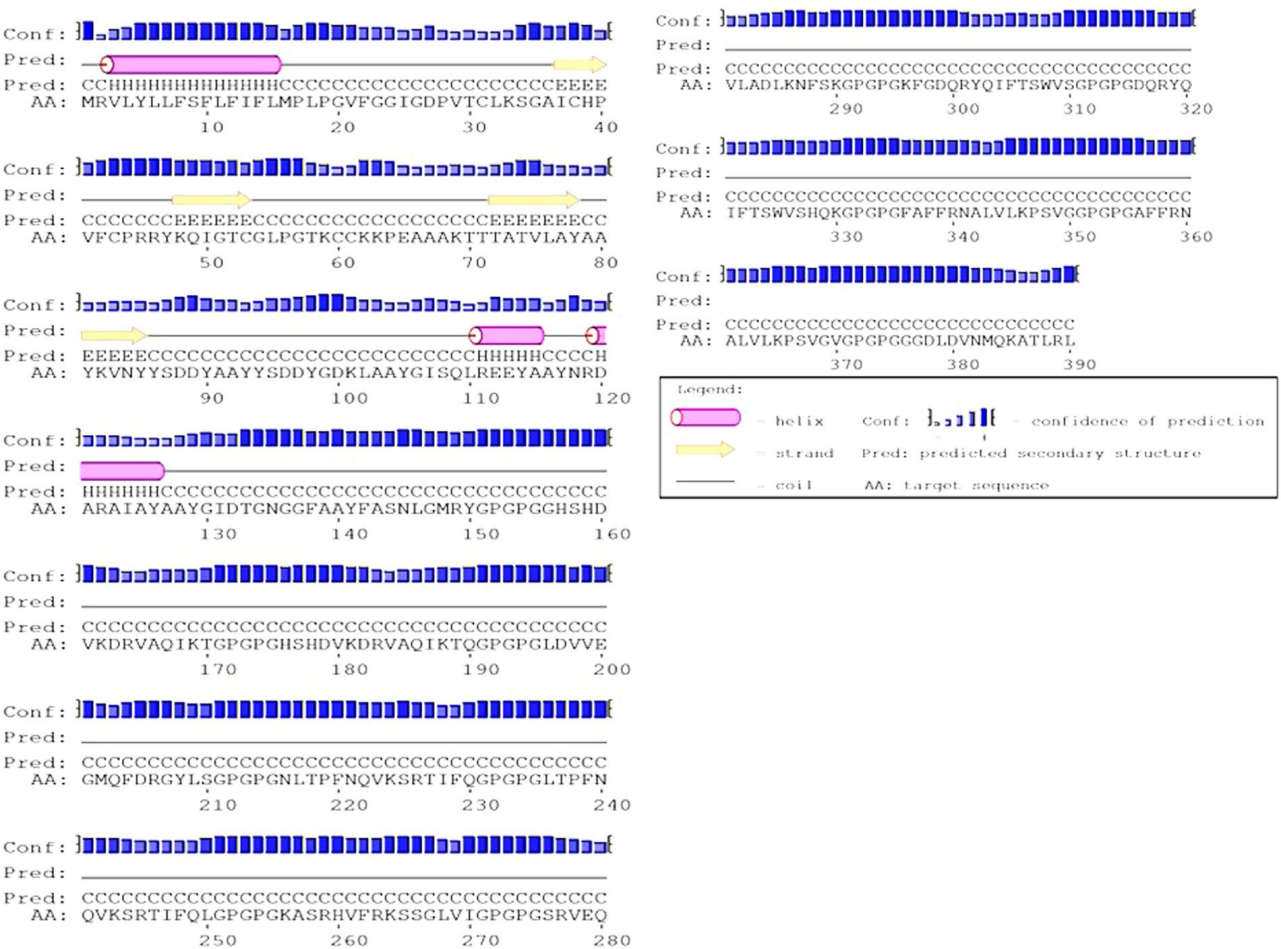
PSIPRED web tool predicted secondary structure elements. The structure was divided as 12.9% β-strand, 35.8% α-helix, and 51.3% coils.

A web server PSIPRED was used to predict the secondary structure, shown in Fig. 4, of the vaccine protein. The forecasted secondary structure elements include 35.8% α-helix, 12.9% β-sheet and 51.3% coils.

### 3D structure modelling and refinement

**Figure 5:**
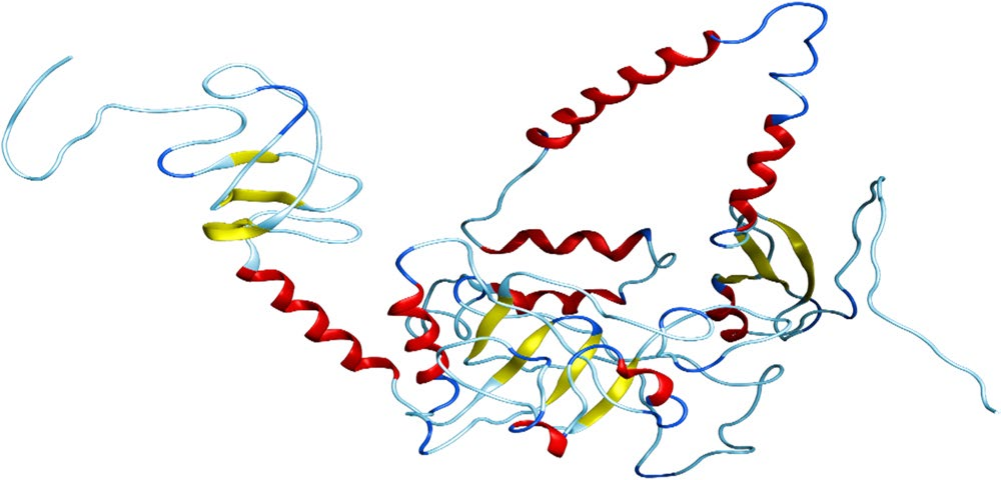
3D structure of the final vaccine modelled by using multi-templates. The respective elements such as loops are given in sky blue colour, helices are in red while beta-sheets are coloured yellow.

3-dimensional structure of the multi-epitope final vaccine was modelled by using RaptorX web tool. The final structure obtained from this server has been shown in Fig. 5. Multi-template modelling predicted the 3D structure in 3 domains. For each domain, different templates were utilized. Templates with accession numbers 1FD3, 2LXO for domain-1, 1IOK, 5CDI, 3RTK, 1GRL, 1WE3 for domain-2 while 5MZ5, 2GOK, 4E57, 3B3J and 4WA0 were used for domain-3 modeling. It was found that 3RTK with *p*-value 7.38e-05, attained score 83 and 18% of identity. Galaxy Refine tool was utilized then to refine the selected model-1 which was then evaluated using RMSD (0.442), MolProbity (2.001), Ramachandran plot (93.3), GDT-HA (0.9365), Poor rotamers (0.3) and clash score (11.2). YASARA energy minimization tool was used to further improve the quality of the vaccine structure.

### 3D structure validation

**Figure 6:**
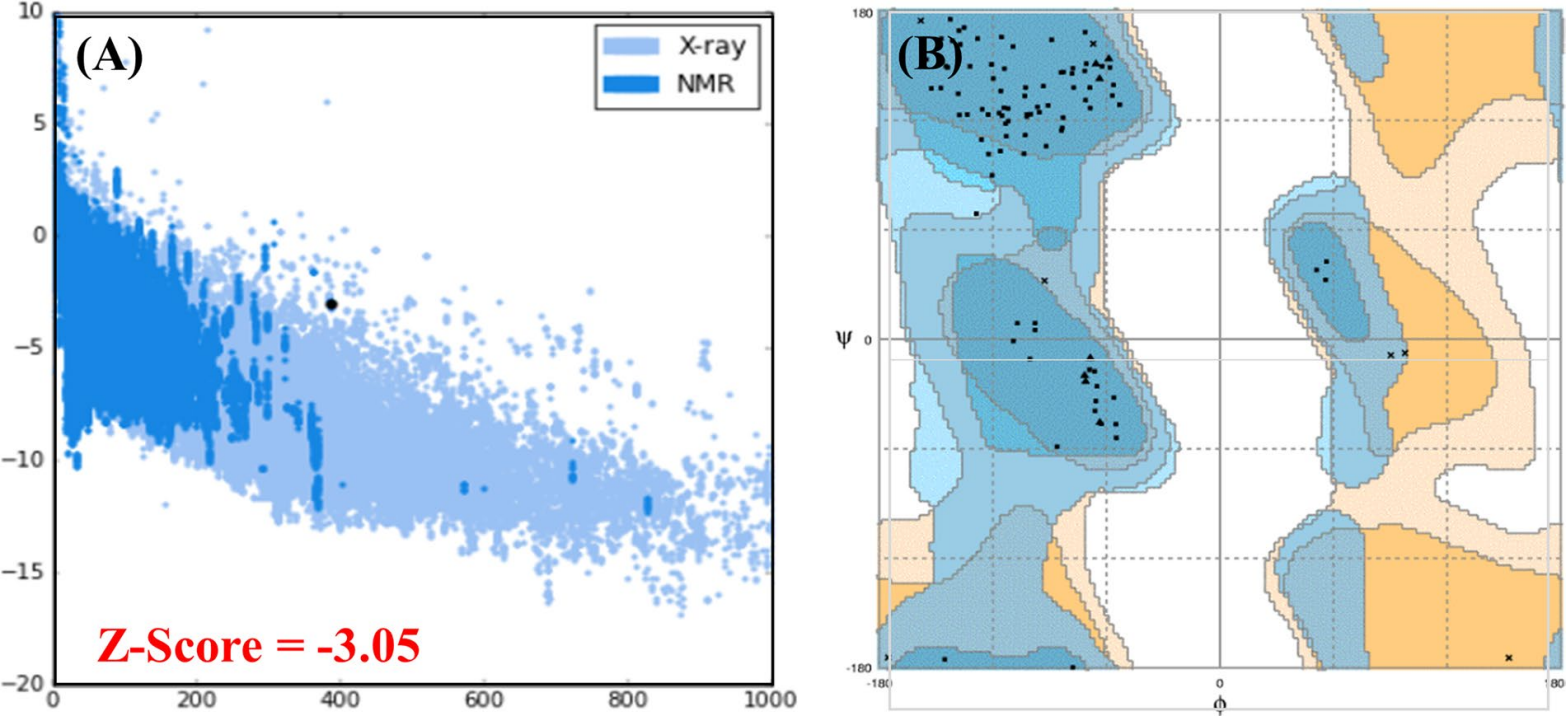
Ramachandran plot and PROSA-web server analysis are given in the above figure. The Z-score (−3.05) predicted by the PROSA-web is given in panel (A) while the distribution of each amino acid in the favoured, allowed and disallowed region are shown by using Ramachandran plot in the panel (B). Overall 82.5% residues were reported to be in favoured region, 13.9% in allowed while 3.6% were reported to be in disallowed region respectively.

Validity of the refined 3-dimensional modelled structure of the multi-epitope vaccine protein was carried out by using RAMPAGE, which verified that 82.5% residues of the vaccine are in the favoured region, 13.9% in allowed while only 3.6% in outlier area. ProSA-web further assessed the quality of the model, which reported the Z-score of −3.05 while 86.3% confidence was reported by the ERRAT server. The plots obtained from RAMPAGE server and PROSA-web are given in Fig. 6.

### Interaction analysis of the vaccine with TLR receptors

**Figure 7:**
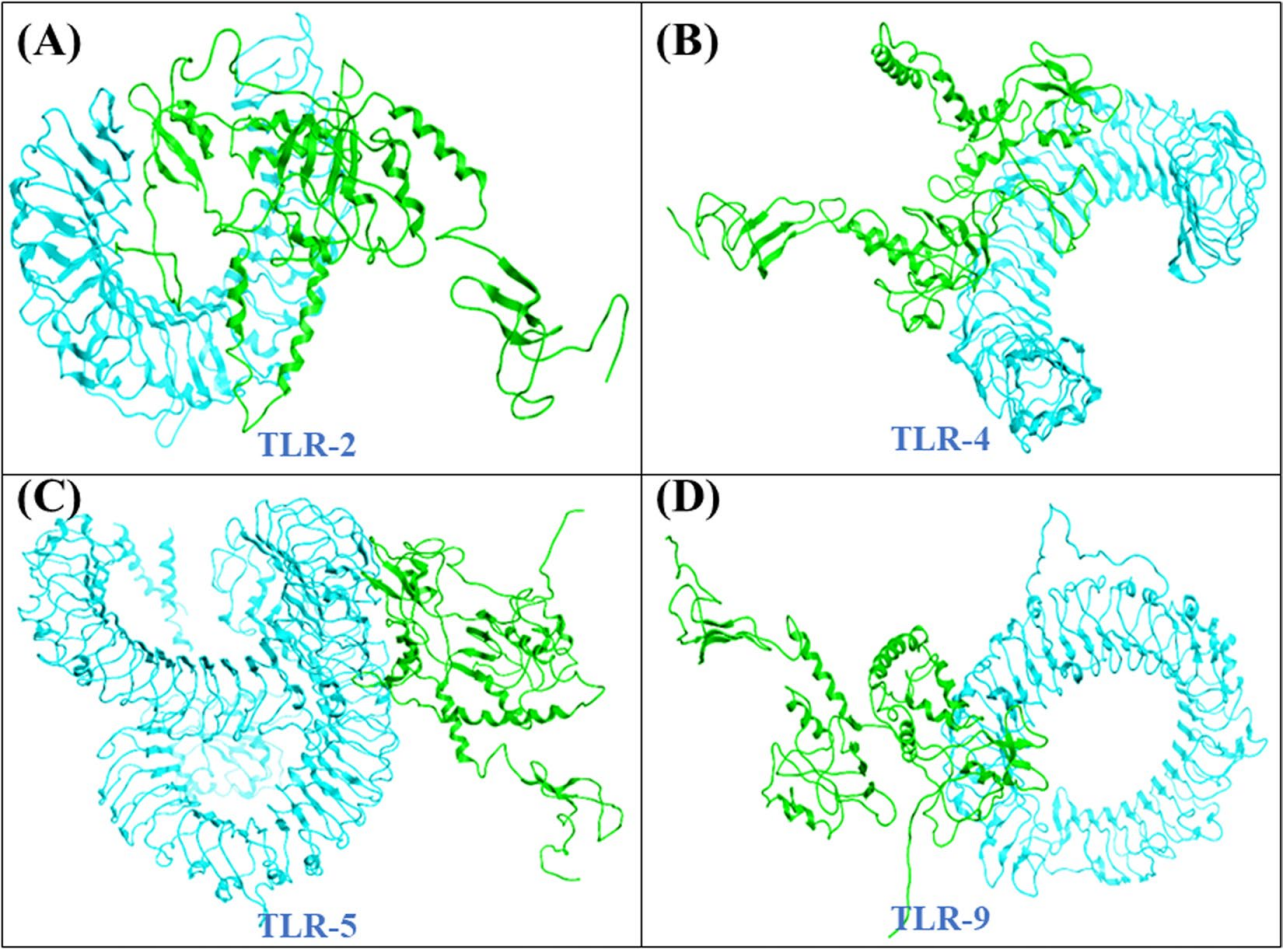
Refined docked complexes of all the TLRs with the vaccine construct. Docking of the final vaccine against each receptor is given in their respective panels. (**A**) TLR-2, (**B**) TLR-4, (**C**) TLR-5 and (**D**) TLR-9. The magenta colour is showing each receptor while green colour is the final vaccine structure.

Multiple TLR receptors (TLR-2, 4, 5 and 9) were used as receptors and are given in Fig. 7. Patchdock server reported the top ten interaction models ranked by geometry of the protein’s surface and electrostatic complementarity. Refinement and rescoring of the top complexes was performed on FireDock web tool which reported the best model with electrostatic interactions (6.12), Van Der Waals associations (−15.09), atomic contact energy (1.05), and binding free energy (−11.97).

### MD simulation of TLRs-vaccine complexes

**Figure 8:**
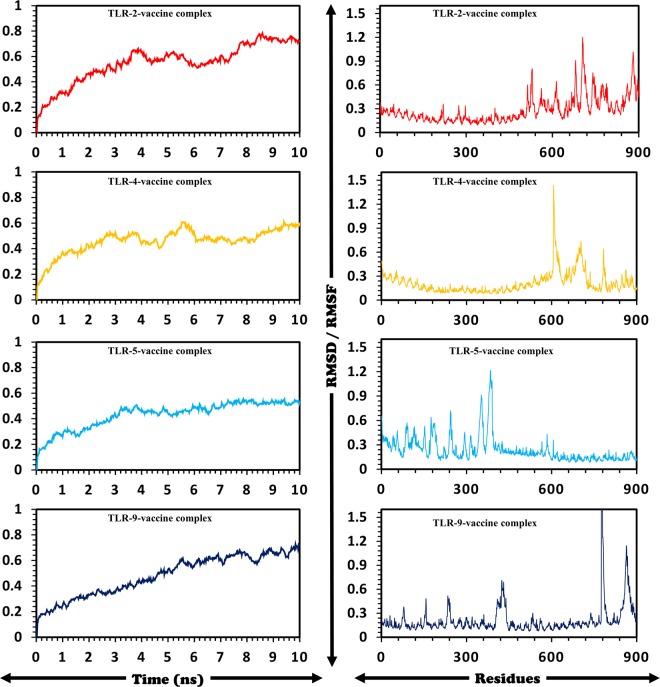
Residual stability and fluctuations i-e (RMSDs) and RMSFs for all the systems are given. Ten nanoseconds simulations were carried out. The left panel (RMSDs) while RMSFs are given in the right panel.

Complexes of final vaccine construct with the selected receptors (TLR-2, 4, 5 and 9) were subjected to MD simulations to calculate the residual stability and fluctuations. RMSD was evaluated to quantify each system’s stability while RMSF was evaluated as shown in Fig. 8 for residual fluctuation. Ten nanoseconds simulations for all the systems were established, which reported variable RMSDs such as 0.7 nm (TLR-2), 0.7 nm (TLR-5), 0.5–0.6 nm (TLR-4) and 0.7 nm for TLR-9. Residual fluctuation (RMSF) was observed within the allowed range with the exception of a few greater fluctuating residues.

### Codon optimization and *in-silico* vaccine expression

**Figure 9:**
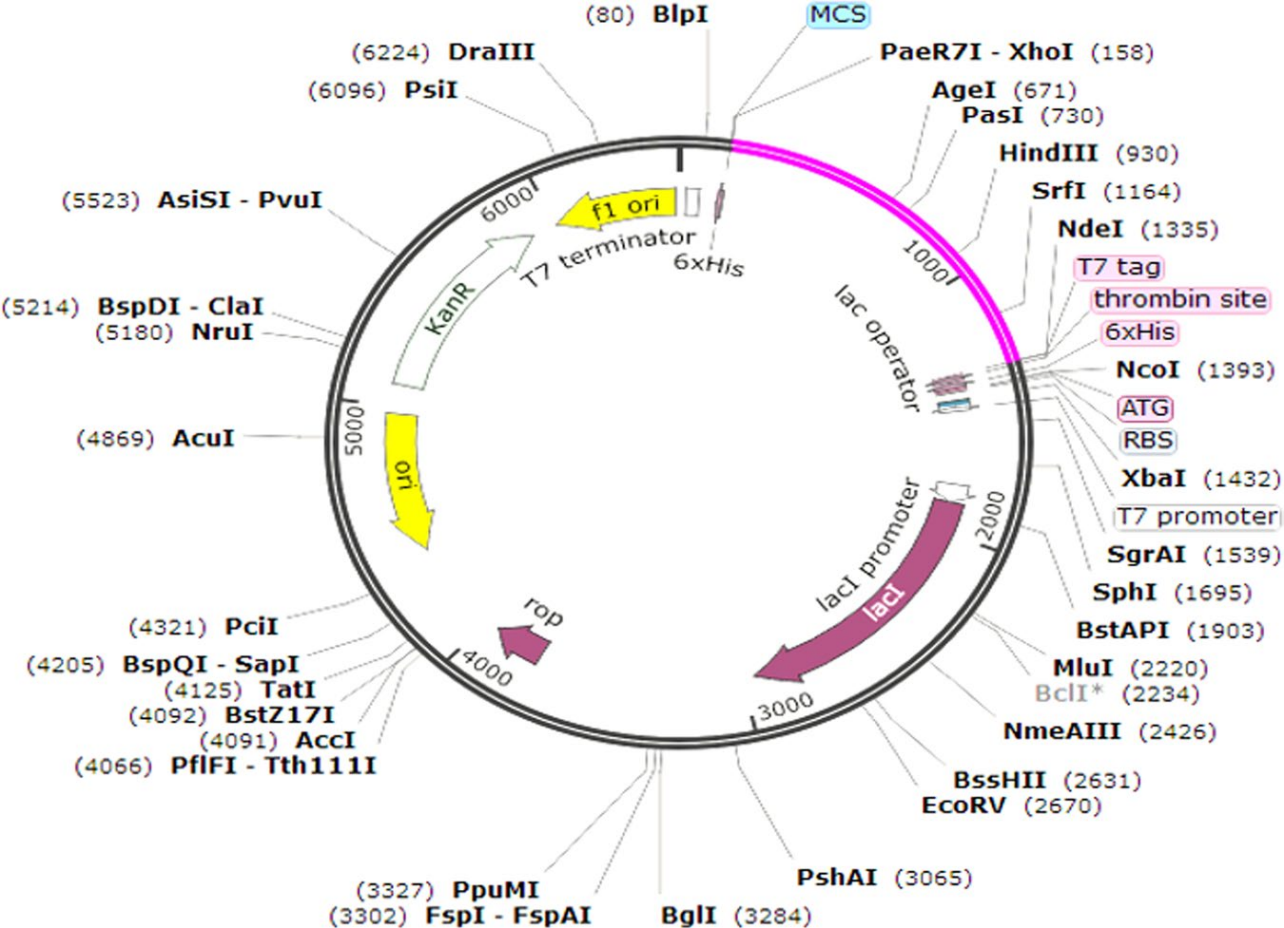
Structure of *In-silico* vector pET28a (+) construct including vaccine along with the vector and restriction sites. The vaccine sequence is highlighted in pink colour.

Herein, Jcat, was utilized to quantify the expression level of the multi-epitopes vaccine *Escherichia coli* (K12 strain). A total of 1170 nucleotides were used as input. Codon optimization was performed and Codon Adaptation Index (CAI) was calculated which was found to be 0.95 with GC contents of 55%. These results indicate the better expression of the final multip-epitope vaccine in *E*. *coli* (K-12 strain). It has been reported that 35% to 70% GC contents for better expression is required. Restriction sites (NdeI and XhoI) were added 5′ and 3′ ends and cloning of the optimized nucleotide sequence in the pET28a (+) vector was performed. The overall construct of this vaccine along with the vector and restriction sites are given in Fig. 9.

### Immune simulation

**Figure 10:**
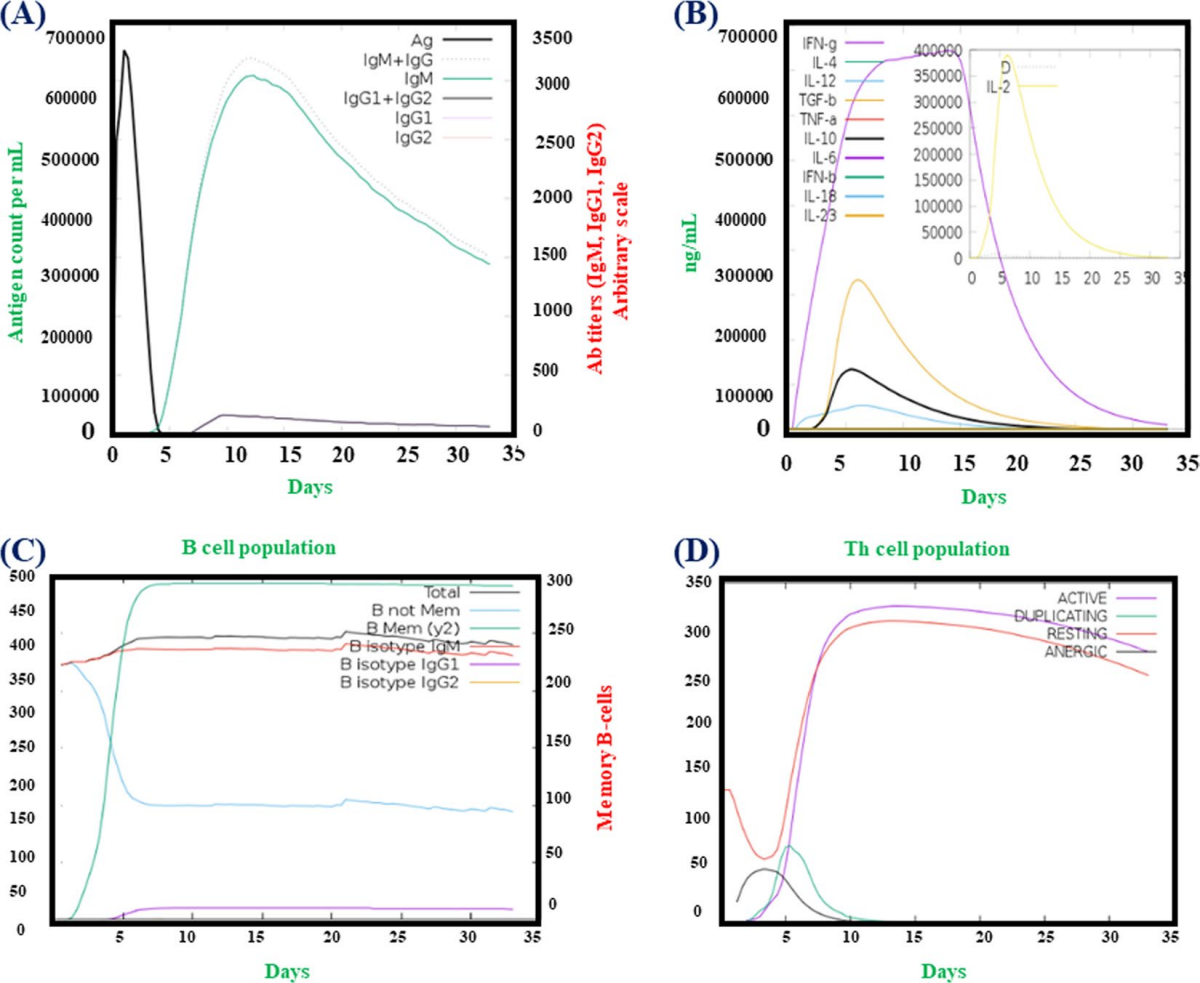
C-ImmSim presentation of an *in silico* immune simulation with the chimeric peptide. (**A**) Antibodies production (the black vertical lines is antigen. (**B**) Cytokines level, (**C**,**D**) shows the B and T-cell population respectively.

It was found that results from the C-ImmSim server are compatible with prior experimental studies’ reported the immune responses in *H*. *pylori* infections. Figure 10 shows the figures obtained from C-ImmSim server. Figure 10(A,C) show that the main reaction triggers the production of IgG and IgM antibodies, while the secondary reaction indicates enhanced levels of IgG1 + IgG2, IgM, and IgG + IgM (B-cell populations) antibodies. Earlier screening of *H*. *pylori* patients was reported to have IgM and IgG antibodies^55^. Furthermore, IFN- γ, Th1 and Th2 responses were also tested. Previously studies revealed that the *H*. *pylori* infections are characterized by Th1, Th2 and IFN- γ and both Th1 and Th2 responses are required for the protection^56^. Herein, Fig. 10(B,C), the IFN- γ concentration and TH cell population are reported to be high. Thus, these findings show that our vaccine design could effectively trigger the immune response and provide the basis for immunity against *H*. *pylori*-associated infections.

## Discussion

To control infectious diseases, the use of antibiotics is not only expensive but is also precariously resulting in the continuous generation of resistant microbes. Alternatively, vaccination can be effectively applied to a vast population to prevent infections. Conversely, to the classical approaches for vaccinations, today’s cutting-edge research has made possible the generation of “subunit vaccines” which constitutes of the particular pathogenic protein sequences of the microbes and are fully capable of effective stimulation of immune response. Due to the availability of comprehensive information about the genomes and proteome of the microbes designing novel and efficient subunit vaccines has become more practically possible. As conventional vaccine development approaches are now almost outdated due to their low effectiveness and high costs in terms of budget and time. Designing vaccines via immunoinformatics approach is comparatively stable, safe, inexpensive, specific and more effective.

Utilizing the cutting-edge immune-informatics approach proteome of the *H*. *pylori* was exploited to identify the target sequences for creating the subunit vaccine against the *H*. *pylori*. As previously documented, the most recognizable toxic factors in *H*. *pylori* infections are cag, PaI, VacA, cagþ, dupA, OipA, dupA and BabA. To assess the immunogenic potency of these proteins comprehensive analysis was carried out using online servers, and online tools were applied to generate a potent subunit vaccine design. Additionally, the B-lymphocyte and T-lymphocyte epitopes were determined from the chosen protein sequences. Generally, the T cell receptors (TCR) on T-lymphocytes are responsible, to generate the immune response, after activated by the antigen-presenting cells (APCs) or MHC bound antigens, and these two molecules MHC-I and MHC-II. The immune-informatics scrutiny of the *H*. *pylori* proteome determines, that the proposed vaccine protein covers a comprehensive number of high affinities MHC Class I, II and B-cell linear epitope based on physio-chemical property and structural features. And the generation of plasma and memory B cells provides future protection from specific antigens or pathogen-associated antigens. Here our final proposed vaccine design is composed of significant MHC I & II binding epitopes assisted by the adjuvant improves the host immunity. To counter the allergic response to the designed vaccine, the involved key parameters were carefully assessed. PSIPRED v3.3 and RaptorX servers were utilized for the SS and 3D structures accordingly. To further refine the 3D structure GalaxyRefine server was utilized and the refined 3D model was additionally validated utilizing multiple servers for its quality. To assess the interactions among TLR-2, TLR-4, TLR-5, and TLR-9 and the designed vaccine, docking was performed, and for the stability of docking complexes, MD simulations were applied.

For maximized expression and optimization of codons, Jcat software was applied and K12 strain of *E. coli* was used. The GC contents and CAI calculated by Jcat indicated high level expression, the software validation confirmed high solubility of the proposed vaccine with required expression in *E. coli*. Disulfide bonds were used in order to provide stability to vaccine.

Immunoinformatics methods used in this scientific study validated the stability, effectiveness and high-level expression of vaccine protein in *E. coli* host. Furthermore, the use of agent-based model predicted the accurate response upon the injection of the vaccine sequence. The vaccine is specific, non-allergenic, antigenic and can effectively control *H*. *pylori* infections, further clinical trials are required to check the efficacy of vaccine.

## Conclusion

One of the major causes of gastric disorder is *Helicobacter Pylori*. This scientific study used immunoinformatics to design a novel and potent multi-epitope vaccine against *H*. *pylori* to design novel multi-epitope vaccine, which could provide both types of immunity. Molecular docking, thermodynamics stability profiling, *in-silico* expression and an agent-based modelling tool to verify the stability, expression and immune response reaction provoked by the final vaccine. The result we got increases the experimental validity and can be helpful in designing of an effective vaccine against *H*. *pylori* infection.

## Supplementary information

Supplementary Information

## References

[1] Floch P, Mégraud F, Lehours P (2017). Helicobacter pylori strains and gastric MALT lymphoma. Toxins.

[2] Peek RM, Blaser MJ (2002). Helicobacter pylori and gastrointestinal tract adenocarcinomas. Nature Reviews Cancer.

[3] Suerbaum S, Michetti P (2002). Helicobacter pylori infection. New England Journal of Medicine.

[4] Atherton JC, Blaser MJ (2009). Coadaptation of Helicobacter pylori and humans: ancient history, modern implications. The Journal of clinical investigation.

[5] Backert S, Tegtmeyer N (2017). Type IV secretion and signal transduction of Helicobacter pylori CagA through interactions with host cell receptors. Toxins.

[6] Khatoon J, Prasad K, Prakash Rai R, Ghoshal U, Krishnani N (2017). Association of heterogenicity of Helicobacter pylori cag pathogenicity island with peptic ulcer diseases and gastric cancer. British journal of biomedical science.

[7] Merino E, Flores‐Encarnación M, Aguilar‐Gutiérrez GR (2017). Functional interaction and structural characteristics of unique components of Helicobacter pylori T4 SS. The FEBS journal.

[8] Yuan X-y (2017). Helicobacter pylori with East Asian-type cagPAI genes is more virulent than strains with Western-type in some cagPAI genes. Brazilian journal of microbiology.

[9] Censini S (1996). cag, a pathogenicity island of Helicobacter pylori, encodes type I-specific and disease-associated virulence factors. Proceedings of the National Academy of Sciences.

[10] Yamakawa A (2002). Correlation between Variation of the 3′ Region of the cagA Gene in Helicobacter pylori and Disease Outcome in Japan. The Journal of Infectious Diseases.

[11] Argent RH (2004). Determinants and consequences of different levels of CagA phosphorylation for clinical isolates of Helicobacter pylori. Gastroenterology.

[12] Yamaoka Y (1999). Relationship between the cagA 3′ repeat region of Helicobacter pylori, gastric histology, and susceptibility to low pH. Gastroenterology.

[13] Yamaoka Y (2002). Importance of Helicobacter pylori oipA in clinical presentation, gastric inflammation, and mucosal interleukin 8 production. Gastroenterology.

[14] Yamaoka Y (2006). Helicobacter pylori outer membrane proteins and gastroduodenal disease. Gut.

[15] Yamaoka Y (2002). Helicobacter pylori infection in mice: Role of outer membrane proteins in colonization and inflammation. Gastroenterology.

[16] Yoshida H (2002). The Evaluation of Putative Virulence Factors of Helicobacter pylori for Gastroduodenal Disease b Use of a Short-Term Mongolian Gerbil Infection Model. The Journal of Infectious Diseases.

[17] Gerhard M (1999). Clinical relevance of the Helicobacter pylori gene for blood-group antigen-binding adhesin. Proceedings of the National Academy of Sciences.

[18] Rad R (2002). The Helicobacter pylori Blood Group Antigen-Binding Adhesin Facilitates Bacterial Colonization and Augments a Nonspecific Immune Response. The Journal of Immunology.

[19] Ki M-R (2014). Role of vacuolating cytotoxin VacA and cytotoxin-associated antigen CagA of Helicobacter pylori in the progression of gastric cancer. Molecular and Cellular Biochemistry.

[20] Nürnberger T, Brunner F, Kemmerling B, Piater L (2004). Innate immunity in plants and animals: striking similarities and obvious differences. Immunological Reviews.

[21] Netea MG, Van der Graaf C, Van der Meer JWM, Kullberg BJ (2004). Toll-like receptors and the host defense against microbial pathogens: bringing specificity to the innate-immune system. Journal of Leukocyte Biology.

[22] Torok AM, Bouton AH, Goldberg JB (2005). Helicobacter pylori Induces Interleukin-8 Secretion by Toll-Like Receptor 2- and Toll-Like Receptor 5-Dependent and -Independent Pathways. Infection and Immunity.

[23] Ding S-Z, Torok AM, Smith MF, Goldberg JB (2005). Toll-like Receptor 2-Mediated Gene Expression in Epithelial Cells During Helicobacter pylori Infection. Helicobacter.

[24] Peek RM (2004). Helicobacter pylori Flagellin Evades Toll-Like Receptor 5-Mediated Innate Immunity. The Journal of Infectious Diseases.

[25] Ishihara S (2004). Essential Role of MD-2 in TLR4-Dependent Signaling during Helicobacter pylori-Associated Gastritis. The Journal of Immunology.

[26] Sutton, P. & Boag, J. M. Status of vaccine research and development for Helicobacter pylori. *Vaccine*, 10.1016/j.vaccine.2018.01.001 (2018). 10.1016/j.vaccine.2018.01.001 PMC6892279 29627231

[27] Larsen JEP, Lund O, Nielsen M (2006). Improved method for predicting linear B-cell epitopes. Immunome research.

[28] Khan A (2018). Computational identification, characterization and validation of potential antigenic peptide vaccines from hrHPVs E6 proteins using immunoinformatics and computational systems biology approaches. PloS one.

[29] Ali A (2019). Immunoinformatic and systems biology approaches to predict and validate peptide vaccines against Epstein–Barr virus (EBV). Scientific Reports.

[30] Sakharkar, K. R., Sakharkar, M. K. & Chandra, R. *Post-Genomic Approaches in Drug and Vaccine Development*. Vol. 5 (River Publishers, 2015).

[31] Larsen MV (2007). Large-scale validation of methods for cytotoxic T-lymphocyte epitope prediction. BMC bioinformatics.

[32] EL-Manzalawy Y, Dobbs D, Honavar V (2008). Predicting linear B-cell epitopes using string kernels. Journal of Molecular Recognition: An Interdisciplinary Journal.

[33] Ponomarenko J (2008). ElliPro: a new structure-based tool for the prediction of antibody epitopes. BMC bioinformatics.

[34] Livingston B (2002). A rational strategy to design multiepitope immunogens based on multiple Th lymphocyte epitopes. The Journal of Immunology.

[35] Saadi M, Karkhah A, Nouri HR (2017). Development of a multi-epitope peptide vaccine inducing robust T cell responses against brucellosis using immunoinformatics based approaches. Infection, Genetics and Evolution.

[36] Eslami, M., Nezafat, N., Negahdaripour, M. & Ghasemi, Y. Computational approach to suggest a new multi-target-directed ligand as a potential medication for Alzheimer’s disease. *Journal of Biomolecular Structure and Dynamics*, 1–15 (2019). 10.1080/07391102.2018.1564701 30689517

[37] Dorosti H (2019). Vaccinomics approach for developing multi-epitope peptide pneumococcal vaccine. Journal of Biomolecular Structure and Dynamics.

[38] Saha S, Raghava G (2006). AlgPred: prediction of allergenic proteins and mapping of IgE epitopes. Nucleic acids research.

[39] Cheng J, Randall AZ, Sweredoski MJ, Baldi P (2005). SCRATCH: a protein structure and structural feature prediction server. Nucleic acids research.

[40] Gasteiger, E. *et al*. In *The proteomics protocols handbook* 571–607 (Springer, 2005).

[41] McGuffin LJ, Bryson K, Jones DT (2000). The PSIPRED protein structure prediction server. Bioinformatics.

[42] Källberg M (2012). Template-based protein structure modeling using the RaptorX web server. Nature protocols.

[43] Ko J, Park H, Heo L, Seok C (2012). GalaxyWEB server for protein structure prediction and refinement. Nucleic acids research.

[44] Krieger E, Vriend G (2014). YASARA View—molecular graphics for all devices—from smartphones to workstations. Bioinformatics.

[45] Wiederstein M, Sippl MJ (2007). ProSA-web: interactive web service for the recognition of errors in three-dimensional structures of proteins. Nucleic acids research.

[46] Colovos C, Yeates TO (1993). Verification of protein structures: patterns of nonbonded atomic interactions. Protein science.

[47] Lovell SC (2003). Structure validation by Cα geometry: φ, ψ and Cβ deviation. Proteins: Structure, Function, and Bioinformatics.

[48] Schneidman-Duhovny D, Inbar Y, Nussinov R, Wolfson HJ (2005). PatchDock and SymmDock: servers for rigid and symmetric docking. Nucleic acids research.

[49] Andrusier N, Nussinov R, Wolfson HJ (2007). FireDock: fast interaction refinement in molecular docking. Proteins: Structure, Function, and Bioinformatics.

[50] Pearlman DA (1995). AMBER, a package of computer programs for applying molecular mechanics, normal mode analysis, molecular dynamics and free energy calculations to simulate the structural and energetic properties of molecules. Computer Physics Communications.

[51] SalomonFerrer R, Case DA, Walker RC (2013). An overview of the Amber biomolecular simulation package. Wiley Interdisciplinary Reviews: Computational Molecular Science.

[52] Roe DR, Cheatham TE (2013). PTRAJ and CPPTRAJ: software for processing and analysis of molecular dynamics trajectory data. Journal of chemical theory and computation.

[53] Rapin N, Lund O, Castiglione F (2011). Immune system simulation online. Bioinformatics.

[54] Doytchinova IA, Flower DR (2007). VaxiJen: a server for prediction of protective antigens, tumour antigens and subunit vaccines. BMC bioinformatics.

[55] Wilson KT, Crabtree JE (2007). Immunology of Helicobacter pylori: insights into the failure of the immune response and perspectives on vaccine studies. Gastroenterology.

[56] Taylor JM, Ziman ME, Canfield DR, Vajdy M, Solnick JV (2008). Effects of a Th1- versus a Th2-biased immune response in protection against Helicobacter pylori challenge in mice. Microbial pathogenesis.

