# Supplementary material for: Immunoinformatics approaches to explore Helicobacter Pylori proteome (Virulence Factors) to design B and T cell multi-epitope subunit vaccine
Source: Sci Rep. 2019 Sep 16;9:13321. doi: 10.1038/s41598-019-49354-z (PMC6746805; doi:10.1038/s41598-019-49354-z)
Supplement: Supplementary file 1 — Supplementary Information [file 41598_2019_49354_MOESM1_ESM.docx]

**Supplementary Materials**

**Immunoinformatics approaches to explore *Helicobacter Pylori* proteome (Virulence Factors) to design B and T cell multi-epitope subunit vaccine**

Mazhar Khan^1^, Shahzeb Khan^2^, Asim Ali^1^, Hameed Akbar^4^, Abrar Muhammad Sayaf^2^, Abbas Khan^2, 3^, Dong-Qing Wei^3*^

^1^The CAS Key Laboratory of Innate Immunity and Chronic Diseases, Hefei National Laboratory for Physical Sciences at Microscale, School of Life Sciences, CAS Center for Excellence in Molecular Cell Science, University of Science and Technology of China (USTC), Collaborative Innovation Center of Genetics and Development, Hefei, 230027, Anhui, China.

^2^Centre for Biotechnology and Microbiology, University of Swat, Swat, Khyber Pakhtunkhwa, Pakistan.

^3^Department of Bioinformatics and Biological Statistics, School of Life Sciences and Biotechnology, Shanghai Jiao Tong University, Shanghai 200240, P.R China.

^4^Laboratory of Cellular Dynamics, School of Life Sciences, University of Science and Technology of China (USTC).

Mazhar Khan

[mazharsw@mail.ustc.edu.cn](mailto:mazharsw@mail.ustc.edu.cn)

Shahzeb Khan

[khanshahxeb173@gmail.com](mailto:khanshahxeb173@gmail.com)

Asim Ali

[Asim21pk@gmail.com](mailto:Asim21pk@gmail.com)

Hameed Akbar,

Hameedakbar4181@mail.ustc.edu.cn

Abrar Muhammad Sayaf

[Chemdr07@gmail.com](mailto:Chemdr07@gmail.com)

Abbas Khan

[abbaskhan@sjtu.edu.cn](mailto:abbaskhan@sjtu.edu.cn)

Dong-Qing Wei (corresponding author)

[dqwei@sjtu.edu.cn](mailto:dqwei@sjtu.edu.cn)

**Table S1:** Cytotoxic T-Lymphocytes epitopes identified.

| **Protein** | **Residue NO** | **Peptide sequence** | **MHC Binding affinity** | **Rescale binding affinity** | **C-terminal cleavage affinity** | **Transport affinity** | **Prediction score** | **MHC-I Binding** |
| --- | --- | --- | --- | --- | --- | --- | --- | --- |
| **Gi:446963037, chaperonin GroEL** | 7 | FSDSARNLL | 0.3712 | 1.5762 | 0.6218 | 0.8250 | 1.7107 | yes |
|  | **88** | TTTATVLAY | 0.7203 | 3.0584 | 0.9620 | 2.8160 | 3.3435 | yes |
|  | 152 | NSDHNIGKL | 0.2194 | 0.9317 | 0.8385 | 1.0100 | 1.1080 | yes |
|  | 210 | MTAQLDNAY | 0.7529 | 3.1966 | 0.6508 | 3.0680 | 3.4476 | yes |
|  | 213 | QLDNAYILL | 0.2023 | 0.8589 | 0.9539 | 0.9650 | 1.0502 | yes |
|  | 221 | LTDKKISSM | 0.2744 | 1.1652 | 0.7842 | 0.1590 | 1.2908 | yes |
|  | 300 | ISEELGLSL | 0.1333 | 0.5658 | 0.9464 | 0.9210 | 0.7538 | yes |
|  | 351 | QIASTTSDY | 0.3531 | 1.4990 | 0.9512 | 3.0930 | 1.7963 | yes |
|  | 513 | TTEATVHEI | 0.2253 | 0.9566 | 0.8508 | 0.3960 | 1.1041 | yes |
| **Gi:446632395,**  **outer inflammatory protein**  **OipA** | 15 | LHAERNGFY | 0.1278 | 0.5427 | 0.4242 | 2.8970 | 0.7512 | yes |
|  | 80 | DSNKIASRF | 0.1159 | 0.4922 | 0.9662 | 2.2660 | 0.7504 | yes |
|  | 147 | NTDLLINWT | 0.2062 | 0.8756 | 0.0314 | -0.9710 | 0.8318 | yes |
|  | 168 | RVKGLSIFY | 0.1380 | 0.5858 | 0.8687 | 3.4370 | 0.8880 | yes |
|  | 183 | TLDANTLKK | 0.1654 | 0.7023 | 0.9658 | 0.4190 | 0.8681 | yes |
|  | 273 | KVNYYSDDY | 0.2689 | 1.1415 | 0.8007 | 2.9710 | 1.4102 | yes |
|  | 277 | YSDDYGDKL | 0.4390 | 1.8640 | 0.8498 | 0.7470 | 2.0288 | yes |
| **Gi:2498230, Cytotoxicity associated immune-dominant antigen cagA** | 36 | KVDNVVASF | 0.1380 | 0.5860 | 0.9768 | 2.6130 | 0.8632 | yes |
|  | 65 | GISQLREEY | 0.1879 | 0.7979 | 0.9289 | 2.7310 | 1.0738 | yes |
|  | 91 | FIDKSNDLI | 0.2135 | 0.9063 | 0.1925 | 0.2530 | 0.9479 | yes |
|  | 125 | FTSWVSHQK | 0.1566 | 0.6647 | 0.9669 | 0.3530 | 0.8274 | yes |
|  | 182 | RTDQKFMGV | 0.2884 | 1.2244 | 0.8152 | 0.3700 | 1.3652 | yes |
|  | 212 | WLDIFLSFI | 0.2194 | 0.9314 | 0.6385 | 0.2120 | 1.0378 | yes |
|  | 249 | TTDIQGLPP | 0.2120 | 0.9002 | 0.0692 | -0.1040 | 0.9054 | yes |
|  | 284 | GVADIDPNY | 0.1639 | 0.6960 | 0.9685 | 2.7340 | 0.9780 | yes |
|  | 316 | EPEKVSLLY | 0.1555 | 0.6604 | 0.9779 | 2.2810 | 0.9211 | yes |
|  | 418 | LSEKEKEKF | 0.1442 | 0.6125 | 0.2986 | 2.5760 | 0.7860 | yes |
|  | 463 | ITEFNNGDL | 0.1547 | 0.6567 | 0.9209 | 0.8600 | 0.8378 | yes |
|  | 505 | FVDYSNFKY | 0.6991 | 2.9683 | 0.9638 | 2.8280 | 3.2542 | yes |
|  | 669 | NRDARAIAY | 0.2057 | 0.8733 | 0.9332 | 3.1460 | 1.1706 | yes |
|  | 769 | KSDLENSVK | 0.1625 | 0.6898 | 0.2787 | 0.5900 | 0.7611 | Yes |
|  | 785 | VTDKVDNLN | 0.2400 | 1.0190 | 0.0529 | -1.3500 | 0.9594 | Yes |
|  | 834 | NTGKNSELY | 0.6100 | 2.5898 | 0.9445 | 2.6720 | 2.8651 | Yes |
|  | 894 | STEPIYAKV | 0.1867 | 0.7926 | 0.9713 | 0.3430 | 0.9554 | Yes |
|  | 910 | VASPEEPIY | 0.1391 | 0.5905 | 0.5934 | 2.9860 | 0.8288 | Yes |
|  | 964 | LSASPEPIY | 0.2576 | 1.0939 | 0.9139 | 2.9560 | 1.3788 | Yes |
|  | 974 | TIDDLGGPF | 0.1615 | 0.6859 | 0.5831 | 2.6200 | 0.9043 | Yes |
|  | 1015 | VSEAKAGFF | 0.3085 | 1.3100 | 0.0717 | 2.6470 | 1.4531 | Yes |
|  | 1037 | TKKNVMNLY | 0.1299 | 0.5517 | 0.9505 | 3.0050 | 0.8445 | Yes |
|  | 1055 | SLSAKLDNY | 0.2276 | 0.9665 | 0.7782 | 3.1470 | 1.2406 | Yes |
|  | 1129 | YSDSFKFST | 0.2677 | 1.1366 | 0.0424 | -1.0000 | 1.0930 | Yes |
|  | 1150 | FTHFLANAF | 0.1713 | 0.7272 | 0.2691 | 2.4180 | 0.8885 | yes |
|  | 1154 | LANAFSTGY | 0.3152 | 1.3381 | 0.9606 | 2.7820 | 1.6213 | Yes |
| **Gi:15645505, vacuolating cytotoxin autotransporter vacA** | 135 | DMKDAVGTY | 0.1680 | 0.7134 | 0.9777 | 2.7750 | 0.9988 | Yes |
|  | 231 | SKNAEISLY | 0.1459 | 0.6196 | 0.8845 | 2.9870 | 0.9016 | Yes |
|  | 273 | YSTINTSKV | 0.1589 | 0.6745 | 0.7829 | 0.3530 | 0.8096 | Yes |
|  | 277 | NTSKVTGEV | 0.2254 | 0.9571 | 0.9523 | 0.3350 | 1.1167 | Yes |
|  | 372 | KTEIQPTQV | 0.1484 | 0.6303 | 0.8454 | 0.2070 | 0.7674 | Yes |
|  | 415 | LTTNAAHLH | 0.1956 | 0.8307 | 0.0678 | -0.5350 | 0.8141 | Yes |
|  | 416 | TTNAAHLHI | 0.2052 | 0.8712 | 0.6959 | 0.4460 | 0.9979 | Yes |
|  | 456 | RVNNQVGGY | 0.2121 | 0.9005 | 0.9688 | 3.2610 | 1.2088 | Yes |
|  | 478 | GTDTKNGTA | 0.3002 | 1.2744 | 0.5297 | -0.7580 | 1.3160 | Yes |
|  | 484 | GTATFNNDI | 0.1992 | 0.8456 | 0.5317 | 0.4920 | 0.9499 | Yes |
|  | 501 | KVDAHTANF | 0.1437 | 0.6103 | 0.8337 | 2.6520 | 0.8680 | Yes |
|  | 511 | GIDTGNGGF | 0.1393 | 0.5913 | 0.9123 | 2.4090 | 0.8486 | Yes |
|  | 659 | YSQFSNLTI | 0.1945 | 0.8258 | 0.8400 | 0.3840 | 0.9710 | Yes |
|  | 703 | NVDSATGFY | 0.7135 | 3.0293 | 0.8036 | 3.0650 | 3.3031 | Yes |
|  | 731 | LLKAKIIGY | 0.1093 | 0.4640 | 0.9608 | 2.9930 | 0.7578 | Yes |
|  | 793 | SMVNNPDNY | 0.1581 | 0.6711 | 0.4466 | 3.1280 | 0.8944 | Yes |
|  | 893 | RSKDIDTLY | 0.3762 | 1.5971 | 0.9760 | 3.3480 | 1.9109 | Yes |
|  | 916 | LIDSHDAGY | 0.4998 | 2.1219 | 0.8986 | 2.9290 | 2.4031 | Yes |
|  | 958 | TSSLQTLSL | 0.1453 | 0.6169 | 0.9545 | 1.0710 | 0.8136 | Yes |
|  | 986 | NIDSFAQRL | 0.1341 | 0.5693 | 0.9640 | 1.0850 | 0.7682 | Yes |
|  | 1045 | GTSAGVDAY | 0.5717 | 2.4275 | 0.8444 | 2.6680 | 2.6876 | Yes |
|  | 1111 | GSDQSSLNF | 0.4972 | 2.1112 | 0.9169 | 2.2680 | 2.3621 | Yes |
|  | 1123 | LLRDLNQSY | 0.1458 | 0.6189 | 0.9582 | 3.1510 | 0.9202 | Yes |
|  | 1128 | NQSYNYLAY | 0.3870 | 1.6433 | 0.8923 | 3.0810 | 1.9312 | Yes |
|  | 1136 | YSAATRASY | 0.5348 | 2.2705 | 0.5821 | 3.1160 | 2.5136 | Yes |
|  | 1138 | AATRASYGY | 0.1250 | 0.5308 | 0.9481 | 3.1390 | 0.8299 | Yes |
|  | 1194 | ASANVEARY | 0.4364 | 1.8530 | 0.9373 | 3.2670 | 2.1570 | Yes |
|  | 1195 | SANVEARYY | 0.2018 | 0.8567 | 0.5286 | 3.0900 | 1.0905 | Yes |
|  | 1203 | YYGDTSYFY | 0.1700 | 0.7216 | 0.9619 | 2.9540 | 1.0136 | Yes |
|  | 1280 | FASNLGMRY | 0.5205 | 2.2099 | 0.9725 | 2.8550 | 2.4986 | Yes |
